# Normal Muscle Oxygen Consumption and Fatigability in Sickle Cell Patients Despite Reduced Microvascular Oxygenation and Hemorheological Abnormalities

**DOI:** 10.1371/journal.pone.0052471

**Published:** 2012-12-20

**Authors:** Xavier Waltz, Aurélien Pichon, Nathalie Lemonne, Danièle Mougenel, Marie-Laure Lalanne-Mistrih, Yann Lamarre, Vanessa Tarer, Benoit Tressières, Maryse Etienne-Julan, Marie-Dominique Hardy-Dessources, Olivier Hue, Philippe Connes

**Affiliations:** 1 Inserm 665, Université des Antilles et de la Guyane, Pointe-à-Pitre, Guadeloupe, France; 2 Laboratory of Excellence GR-Ex « The red cell : from genesis to death », PRES Sorbonne Paris Cité, Paris, France; 3 Laboratoire ACTES (EA 3596 - Département de Physiologie), Université des Antilles et de la Guyane, Pointe-à-Pitre, Guadeloupe, France; 4 Laboratoire «Réponses cellulaires et fonctionnelles à l'hypoxie» EA2363, Université Paris 13 - PRES Sorbonne Paris Cité, Bobigny, France; 5 Unité Transversale de la Drépanocytose, Centre Hospitalier et Universitaire, Pointe-à-Pitre, Guadeloupe, France; 6 CIC-EC 802 Inserm, Centre Hospitalier Universitaire, Pointe-à-Pitre, Guadeloupe, France; 7 Centre de référence maladies rares pour la drépanocytose aux Antilles-Guyane, Centre Hospitalier et Universitaire, Pointe-à-Pitre, Guadeloupe, France; University of Mississippi, United States of America

## Abstract

**Background/Aim:**

Although it has been hypothesized that muscle metabolism and fatigability could be impaired in sickle cell patients, no study has addressed this issue.

**Methods:**

We compared muscle metabolism and function (muscle microvascular oxygenation, microvascular blood flow, muscle oxygen consumption and muscle microvascular oxygenation variability, which reflects vasomotion activity, maximal muscle force and local muscle fatigability) and the hemorheological profile at rest between 16 healthy subjects (AA), 20 sickle cell-hemoglobin C disease (SC) patients and 16 sickle cell anemia (SS) patients.

**Results:**

Muscle microvascular oxygenation was reduced in SS patients compared to the SC and AA groups and this reduction was not related to hemorhelogical abnormalities. No difference was observed between the three groups for oxygen consumption and vasomotion activity. Muscle microvascular blood flow was higher in SS patients compared to the AA group, and tended to be higher compared to the SC group. Multivariate analysis revealed that muscle oxygen consumption was independently associated with muscle microvascular blood flow in the two sickle cell groups (SC and SS). Finally, despite reduced muscle force in sickle cell patients, their local muscle fatigability was similar to that of the healthy subjects.

**Conclusions:**

Sickle cell patients have normal resting muscle oxygen consumption and fatigability despite hemorheological alterations and, for SS patients only, reduced muscle microvascular oxygenation and increased microvascular blood flow. Two alternative mechanisms can be proposed for SS patients: 1) the increased muscle microvascular blood flow is a way to compensate for the lower muscle microvascular oxygenation to maintain muscle oxygen consumption to normal values or 2) the reduced microvascular oxygenation coupled with a normal resting muscle oxygen consumption could indicate that there is slight hypoxia within the muscle which is not sufficient to limit mitochondrial respiration but increases muscle microvascular blood flow.

## Introduction

Patients with sickle cell disease (SCD) are characterized by anemia and altered blood rheology which may impair blood flow [1], trigger vaso-occlusive crisis, cause tissue ischemia [2], [3], [4] and limit exercise capacity [5].

Few studies investigated baseline microvascular oxygenation at the cerebral level in SCD patients and reported reduced values suggesting a certain degree of chronic cerebral hypoxia [6], [7], [8]. But, it is unknown whether it is also the case in other organs. Callahan et al [9] previously suggested that muscle function could be altered in some patients with SCD. Moreover, it has been proposed [10] that reduced red blood cell (RBC) deformability in rat *cremaster* muscle could severely affects capillary recruitment and tissue oxygenation. Indeed, one may suggest that the reduced RBC deformability in SCD, in association with the other blood rheological abnormalities, could reduce muscle microcirculatory oxygenation, muscle oxygen consumption and impair muscle function in this population.

However, Rodgers et al [11] previously reported high periodic microvascular blood flow oscillations at the skin level of sickle cell patients (*i.e.,* high flowmotion and vasomotion activities) which might be helpful to maintain the microvascular blood flow despite the presence of hemorheological disturbances. Vasomotion is a form of spontaneous localized oscillations induced by spontaneous contraction and relaxation of the smooth muscle components in the small blood vessel walls which generate rhythmic changes in their diameter. Although vasomotion mechanisms are still under debate [12], several studies demonstrated that vasomotion may have beneficial effects on tissue oxygenation [13], [14], [15]. Therefore the hypothesis of a reduced muscle microvascular oxygenation in SCD patients, due to abnormal hemorheology, compensated by a greater vasomotion activity needs to be tested.

Because SCD patients are characterized by wide hemorheological disturbances, we hypothesized that muscle microvascular oxygenation (TOI), muscle oxygen consumption (VO_2_) and muscle function (*i.e.,* maximal force and fatigability during a short local handgrip exercise) should be impaired in patients with sickle cell anemia (SS) or sickle cell-hemoglobin C disease (SC) in comparison with a control group (AA). We also hypothesized that vasomotion activity could be greater in SCD patients in order to limit muscle VO_2_ reduction. Since the level of VO_2_ is critical for tissue survival we tried to identify factors associated with local muscle VO_2_. We demonstrated that sickle cell patients have normal resting muscle oxygen consumption and fatigability despite hemorheological alterations and, for SS patients only, reduced muscle microvascular oxygenation.

## Materials and Methods

### Patients

The study was approved by the Regional Ethics Committee (CPP Sud-Ouest Outre-Mer III, Bordeaux, France). The experiments were performed in accordance with the guidelines set by the Declaration of Helsinki.

Fifty-two Guadeloupean volunteers (Table 1) participated in the study: 16 healthy subjects with no hemoglobinopathy (AA; 9 males and 7 females), 20 patients with sickle cell-hemoglobin C disease (SC; 10 males and 10 females) and 16 patients with sickle cell anemia (SS; 8 males and 8 females).

**Table 1:** Subjects characteristics, hematological and hemorheological parameters in healthy subjects (AA) and patients with sickle cell-hemoglobin C disease (SC) or sickle cell anemia (SS).

	AA	SC	SS
**Age (yrs)**	34.6±12.5	35.5±12.3	32.9±13.5
**MAP (mmHg)**	90.8±9.2	88.9±11.9	84.8±8.3
**SpO_2_ (%)**	99.6±0.7	99.2±1.0	95.8±3.0^†^*
**SATT (mm)**	3.2±1.0	3.1±1.3	2.8±1.4
**Fetal hemoglobin (%)**	0.4±0.6	1.2±0.8	6.8±5.6*^†^
**Hemoglobin S (%)**	–	47.4±0.9	83.8±6.1*
**Hemoglobin C (%)**	–	43.4±1.3	–
**Leukocytes (10^9^/L)**	6.3±2.1	7.4±2.5	11.3±3.2*^†^
**Red blood cells (10^12^/L)**	4.60±0.52	4.35±0.80	2.88±0.43*^†^
**Hemoglobin (g/dL)**	13.5±1.3	11.1±1.2*	8.4±1.1*^†^
**Hematocrit (%)**	42.0±3.4	32.5±2.8*	24.9±4.3*^†^
**Reticulocytes (%)**	1.1±0.5	2.5±1.6	8.4±3.3*^†^
**Platelets count (10^9^/L)**	248±58	311±171	403±119*
**ηb at 225s^−1^ (mPa/s)**	6.3±1.0	7.9±1.2*	6.3±1.6^†^
**EI at 30 Pa**	0.59±0.02	0.43±0.05*	0.34±0.12*^†^
**RBC aggregation index (%)**	64.6±7.2	43.2±9.4*	52.5±10.6*^†^
**RBC disaggregation threshold (s^−1^)**	146±43	295±128*	394±172*

Values represent mean ± S.D. MAP = mean arterial pressure, SpO_2_ = transcutaneus oxygen saturation, SATT = skin+adipose tissue thickness, ηb = blood viscosity, EI = Elongation Index (*i.e.,* RBC deformability). Different from AA (*p<0.05); different from SC (^†^p<0.05).

Both SS and SC patients recruited are regularly followed by the Sickle Cell Unit of the Academic Hospital of Pointe-à-Pitre (Pointe-à-Pitre, Guadeloupe). All participants were over 18 yrs and were Afro-Caribbean native from Guadeloupe. SCD patients were in clinical steady state at the time of the study (*i.e.,* without vaso-occlusive crisis, acute medical complication or blood transfusion/phlebotomies within the last 3 months). Exclusion criteria for all subjects were recent infectious episode (in the last month), ß-thalassemia, stroke or cerebral vasculopathy history, pregnancy or breast-feeding. Patients taking medication that could affect the hemorheological parameters studied, such as Hydroxyurea, were excluded. All participants received verbal and written explanation of the objectives and procedures of the study and subsequently provided written informed consent.

### Protocol

For each participant, a physician from the Sickle Cell Unit (Guadeloupe) performed a clinical examination with transcutaneus oxygen saturation (SpO_2_) and blood pressure measurements. The mean arterial pressure was calculated as follows: 1/3 systolic pressure +2/3 diastolic pressure. Venous blood was sampled in EDTA tubes from the antecubital vein of the non-dominant arm to perform hematological and hemorheological (*i.e.,* blood viscosity, RBC deformability, RBC aggregation and disaggregation properties) measurements. Then, healthy subjects and SCD patients were placed in a normalized sitting position (thighs-trunk angle of 110° and forearm at heart level to avoid venous pooling of the blood) for 10 min prior the near-infrared spectroscopy (NIRS, NIRO-200, Hamamatsu Photonics, Hamamatsu City, Japan) measurement. The experiment started with a 10-min muscle tissue oxygenation index (TOI) measurement at a sampling frequency of 6 Hz. At the end of this period, three consecutive venous occlusions (50 mmHg) of 30 seconds duration were applied to test microvascular forearm blood flow (mFBF) and muscle VO_2_. One minute of recovery separated each occlusion. Then, subjects performed handgrip exercise test to assess the maximum voluntary contraction (MVC) and local muscle fatigability.

### Near-infrared Spectroscopy and Determination of the Muscle Microvascular Oxygenation (TOI), Microvascular Forearm Blood Flow (mFBF) and Muscle Oxygen Consumption (VO_2_)

NIRS techniques have been described elsewhere [16], [17]. Briefly, NIRS principle is based on the relative transparency of tissue to light in the near-infrared region between 700 nm and 1000 nm, and on the oxygen-dependent absorption changes of hemoglobin. NIRS can be used in sickle cell patients since the near-infrared spectra absorbance of hemoglobin S is similar to the one of normal hemoglobin [18].

The relative concentration changes of oxygenated hemoglobin (ΔHbO_2_), deoxygenated hemoglobin (ΔHHb), total hemoglobin (ΔcHb) and tissue oxyhemoglobin saturation expressed as a tissue oxygenation index (TOI) were measured continuously and simultaneously throughout the experiment at the flexor digitorum superficialis muscle level using two channels of a three-wavelength (775 nm, 810 nm, 850 nm) high temporal resolution NIRS device (NIRO-200, Hamamatsu Photonics, Hamamatsu City, Japan). The center of the NIRS probes was placed on the flexor digitorum superficialis muscle at the proximal third of the forearm. The TOI is given by [ΔHbO_2_/(ΔHHb+ΔHbO_2_)] ×100. The TOI value reflects the microvascular oxygen saturation at the local region of muscle between emission and detection probes. The two probes were firmly attached on the flexor digitorum superficialis muscle with an inter-probes distance of 4 cm by means of a medical adhesive (Hamamatsu Photonics, Massy, France) for measurement of TOI, mFBF and VO_2_.

### Forearm Measurements

#### Adipose tissue thickness

Because adipose tissue thickness (*i.e.,* skin+adipose tissue thickness) is known to affect near infrared spectrometer signals such as oxygenated hemoglobin (ΔHbO_2_), deoxygenated hemoglobin (ΔHHb) and total hemoglobin (ΔcHb), attention was taken to recruit subjects with comparable skinfold thickness [19]. Skinfold thickness was measured between the near infrared spectrometer optodes using a skinfold caliper (Harpenden, West Sussex, U.K) and was divided by 2 to determine the adipose tissue thickness covering the flexor digitorum superficialis muscle.

#### mFBF

The sum of ΔHbO_2_ and ΔHHb reflects the total amount of hemoglobin (ΔcHb), and changes in ΔcHb can be interpreted as changes in blood volume in the tissue [20]. As previously described, mFBF was calculated by evaluating the rate of increase in ΔcHb during venous occlusion [20] within the first seconds of the 30 seconds occlusion. The mFBF was averaged on the data obtained during the three venous occlusions [20]. However, because the hemoglobin concentration is very different between AA, SC and SS groups, we normalized mFBF by dividing the rate of increase in ΔcHb by the hemoglobin concentration for each participant. Normalized concentration changes of ΔcHb were expressed in Δµmol^.^cm^.^dL^.^min**^−^** ^1.^g**^−^** ^1^.

#### VO_2_

Muscle VO_2_ was determined by measuring the rate of increase in ΔHHb within the first seconds of the 30 seconds occlusion [20]. Concentration changes of ΔHHb were expressed in Δµmol^.^cm^.^min**^−^** ^1^. Mean muscle VO_2_ was averaged on the data obtained during the three venous occlusions. The venous occlusion method for muscle VO_2_ measurements used in this study is less reliable than the arterial occlusion method [20]. However, since a short ischemia/hypoxia episode may promote the polymerization of hemoglobin S (HbS) and RBC sickling, hence increasing the risk for a painful vaso-occlusive crisis to occur, we used the venous occlusion method.

### Muscle Force and Fatigability

To test muscle function, maximum voluntary contraction (MVC) force was determined by 3 consecutive MVC interspaced with one-minute recovery. The higher value was considered as being the MVC force (pre-MVC). After a recovery period of 5 min, the subject was asked to perform six intermittent isometric handgrip contraction (5 seconds at 50% MVC) followed by 5 seconds at rest. This protocol is adapted from Hamaoka and colleagues [21]. Ten seconds after the sixth isometric contraction, MVC force was re-determined once (post-MVC). To control the quality of MVC, the root mean square (RMS) obtained with surface electromyography (EMG) was used. The post-MCV was considered maximal only if the RMS of the post-MVC was equal or slightly higher than the RMS of the pre-MVC. This condition was fulfilled for 8 AA subjects, 7 SC and 8 SS patients and statistical analysis of local exercise data was performed on these subgroups.

### Surface Electromyography (EMG) Activity

EMG from the flexor digitorum superficialis muscle was recorded by means of bipolar Ag/AgCl electrodes (Red Dot™, Saint Paul, Canada) with a diameter of 9 mm and an inter-electrode distance of 20 mm. The electrodes were placed on the flexor digitorum superficialis muscle distally after the near infrared spectrometer probes. The reference electrode was placed on the patella. In order to minimize movement artifacts, electrodes and cables were strapped on the subjects using medical hypoallergenic tape. EMG activity was recorded continuously during the exercise cession via a dedicated acquisition system (MP30, Biopac Systems, Santa Barbara, CA, USA). The EMG signals were amplified (1000x), band-pass filtered (30–500 Hz) and sampled at 1000 Hz. The EMG signal amplitude was quantified by the calculation of the RMS. The RMS was calculated over a 1 second period around the maximal value for the MVC (Table 2).

**Table 2:** Muscle force and fatigability (*i.e.,* force decrease) in healthy subjects (AA) and patients with sickle cell-hemoglobin C disease (SC) patients or sickle cell anemia (SS).

	AA	SC	SS
**n (M/F)**	8 (3/5)	7 (3/4)	8 (3/5)
**Pre-MVC (Kg)**	18.2±3.0	14.9±3.5*	13.2±2.5*
**RMS pre-MVC (mV)**	0.60±0.26	0.46±0.10	0.35±0.10
**Post-MVC (Kg)**	14.4±3.0†	12.0±2.3†	11.1±1.8*^†^
**RMS post-MVC (mV)**	0.64±0.30	0.46±0.10	0.39±0.10
**Force decrease (%)**	22.0±6.0	18.4±4.4	17.2±7.7

Values represent mean ± S.D. n (M/F) = sample size (male/female), pre-MVC = maximal voluntary contraction before isometric handgrip exercise, post-MVC = maximal voluntary contraction after isometric handgrip exercise, force decrease = percentage of maximal voluntary contraction decrease after exercise, RMS = root mean square. Post-MVC of SC patients tended to be lower than AA subjects (p = 0.07). Different from AA group (*p<0.05); different from pre-MVC (^†^p<0.05).

### Spectral Analysis of Muscle Microvascular Oxygenation Variability

Muscle microvascular blood flow is variable across the time for a given subject and this variability reflects muscle microvascular flowmotion and vasomotion. In resting condition, the TOI variability mainly reflects blood flow variability since muscle oxygen consumption is constant over the time [22], [23]. Muscle microcirculatory oxygenation exhibits spontaneous oscillations in five frequency components [22], [23]. The Fast Fourier Transform (Welch’s periodogram method) was applied on the TOI signal for the evaluation of the total power of the spectrum in the frequency interval 0.005–2 Hz (*i.e.,* total muscle microcirculatory oxygenation variability) and calculation of the power across 5 band frequencies. The upper limit of 2 Hz was set to include the heart rate frequency, while the lower limit was chosen to include the three lowest frequencies intervals usually observed in tissue oxygenation signals [22], [23], [24]. It has been demonstrated that interval I (0.005–0.02 Hz) reflects nitric oxide metabolism and/or endothelial function [25], [26], [27], interval II (0.02–0.06 Hz) depends on the sympathetic activity of the vessel wall [23], and interval III (0.06–0.20 Hz) corresponds to the myogenic activity [26], For the high oscillatory activities, interval IV (0.20–0.6 Hz) depends on the breathing frequency and interval V (0.6–2 Hz) is under the influence of heart rate and cardiac output [22]. The whole oscillations recorded (*i.e.,* TOI variability or total power of the spectrum) reflect flowmotion. The flowmotion results from the motion of the blood cells and their interaction with the vessel walls. The low frequency domain (intervals I, II and III) corresponds to the vasomotion activity [22], [23]. The power of each interval was also analyzed and expressed in percent contribution of the total power of TOI variability.

### Hematological Parameters

Hematocrit was measured after blood microcentrifugation at 9500 g for 10 min (JOUAN-HEMA-C, Saint Herblain, France). Total counts of leukocytes, platelets and RBCs, percentage of reticulocytes and hemoglobin concentration were determined using a hematology analyzer (Max M-Retic, Coulter, USA).

### Hemorheological Parameters Measurement

Hemorheological parameters were measured within the four hours after sampling and after full re-oxygenation of blood for 10–15 min [28]. Blood viscosity was measured at ≈ 25°C and native hematocrit using a cone-plate viscometer (Brookfield DVII+ with CPE40 spindle) at 225 s**^−^** ^1^. The RBC elongation index values were determined at 30 Pa by laser diffraction analysis (ecktacytometry) and at 37°C, using the Laser assisted Optical Rotational Cell Analyzer (LORCA, RR Mechatronics, Hoorn, The Netherlands). The system calculates an average RBC elongation index. The higher this index, the more deformable the RBCs. RBC aggregation was determined at 37°C via syllectometry (*i.e.,* laser backscatter versus time), using the LORCA (RR Mechtronics, Hoom, The Netherlands), after adjustment of hematocrit to 40%. The system calculates an aggregation index. The RBC disaggregation threshold (*i.e.,* the minimal shear rate needed to prevent RBC aggregation or to break down existing RBC aggregates) was determined using a re-iteration procedure [29]. The RBC disaggregation threshold mainly reflects the RBC aggregate strength while the RBC aggregation index is a measure of the extent of aggregation integrated during a time period of 2 minutes. Hemorheological measurements were performed according to the international guidelines for hemorheological laboratory techniques [28].

### Statistics

All values were expressed as means ± standard deviations. The data were tested for the normality (Kolmogorov-Smirnov test) and homogeneity of variance (Levene test). Physiological, hematological, hemorheological and muscle oxygenation parameters were compared between the three groups using a one-way analysis of variance (ANOVA) followed by a Newman-Keuls post-hoc test. When the rules for parametric test application were not fulfilled, a Kruskal-Wallis test followed by Dunn’s multiple comparison tests was used.

To identify factors associated with muscle VO_2_ (*i.e.,* dependent variable) in AA, SC or SS subjects, we used parametric or non parametric correlations (Pearson or Spearman, respectively) between muscle VO_2_ and the others physiological/biological parameters. Then all variables at p<0.2 were included in a multivariate linear regression models to identify the covariates independently associated with muscle VO_2_. The significance level was defined as p<0.05. Analyses were conducted using SPSS (v. 20, IBM SPSS Statistics, Chicago, IL).

## Results

### Subjects’ Characteristics, Hematological and Hemorheological Parameters

The subjects’ characteristics, hematological and hemorheological parameters are summarized in the Table 1.

Age, mean arterial pressure and skin+adipose tissue thickness were not significantly different between the three groups. SpO_2_ was lower in the SS group compared to both AA and SC groups.

Fetal hemoglobin level, leukocytes and percentage of reticulocytes were significantly higher in the SS group than in both SC and AA groups. RBC count was significantly lower in the SS group compared to the SC and AA groups. Hemoglobin and hematocrit were different between the three groups with AA>SC>SS. The platelets count was higher in the SS group than in the AA group and no difference was observed between SC patients and AA subjects or SS patients.

Blood viscosity was greater in the SC group than in the AA and SS groups. The SS and AA groups were not significantly different regarding blood viscosity. RBC elongation index was significantly different between the three groups in the following order: SS<SC<AA. The RBC aggregation index was different between the three groups such as SC<SS<AA. The RBC disaggregation threshold was higher in the two SCD groups in comparison with AA subjects.

### Muscle Microvascular Oxygenation (TOI), Microvascular Forearm Blood Flow (mFBF) and Muscle Oxygen Consumption (VO_2_)

**Figure 1:**
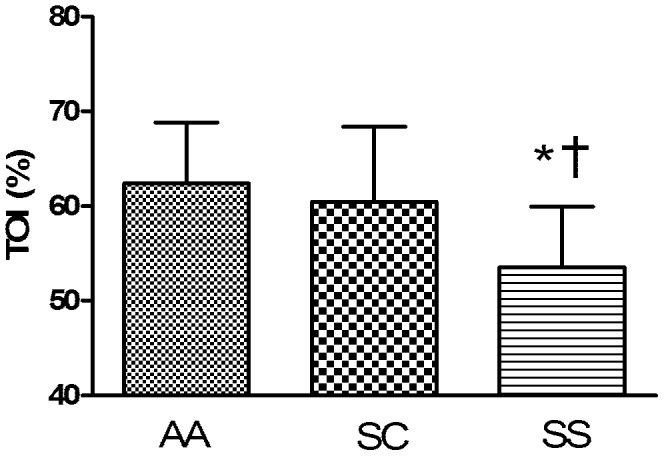
Muscle microvascular oxygen saturation (TOI) at rest in AA, SC and SS groups. Different from AA group (*p<0.05); different from SC group (^†^p<0.05).

**Figure 2:**
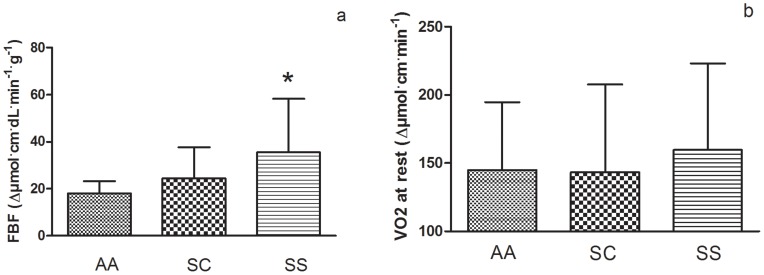
Forearm blood flow at rest (2a) and muscle oxygen consumption at rest (2b) in AA, SC and SS groups.

The muscle TOI (Figure 1) was lower in SS group compared to both AA and SC groups and this was not related to the degree of hemorheological alterations as no significant correlation was found between muscle TOI and hemorheological parameters. mFBF was significantly higher in the SS group than in the AA group, and tended to be higher compared to the SC group (Figure 2a). No statistical difference was observed between the three groups for muscle VO_2_ (Figure 2b).

### Muscle TOI Variability

**Figure 3:**
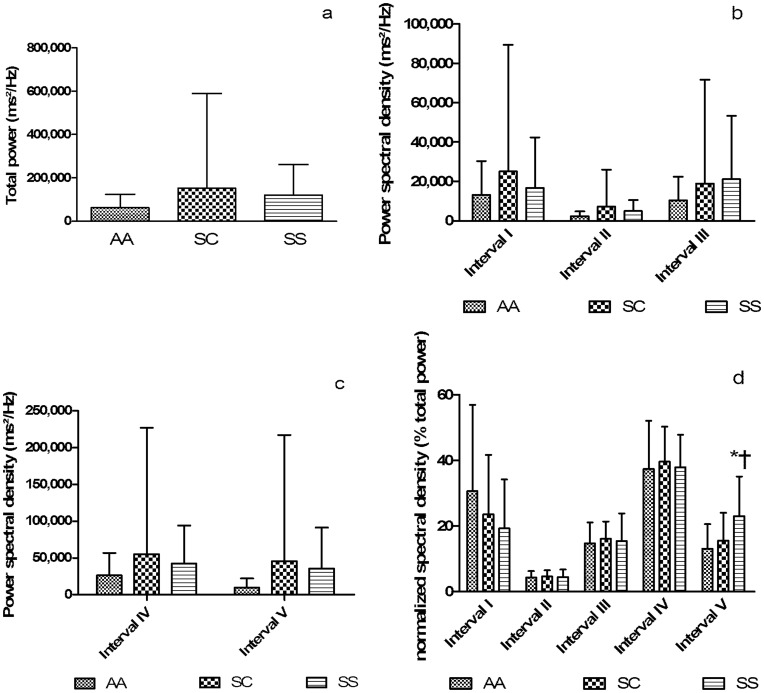
Fast Fourier Transform analysis of TOI signal variability. Total power spectral density (3a, flowmotion activity), power spectral density in interval I, II and III (3b, vasomotion activity), power spectral density in interval IV, V (3c) and normalized spectral density (3d). Interval I = endothelial activity and/or nitric oxide metabolism, interval II = nervous sympathetic activity of the vessel wall, interval III = myogenic activity, interval IV = breathing frequency and interval V heart rate and cardiac output.

**Figure 4:**
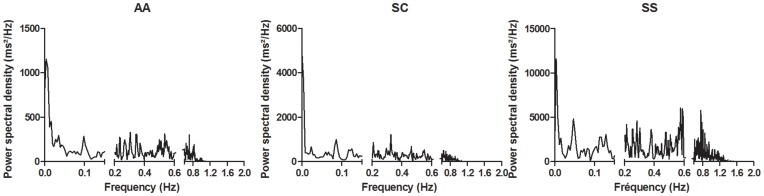
Example of Fast Fourier Transform analysis of TOI signal variability in one AA subject (left), one SC patient (middle) and one SS patient (right). The frequency axis is divided into three parts: vasomotion activity (left), interval IV (middle), and interval V (right). Note that the scale of the Y-axis is different for the three subjects.

The total muscle TOI variability (Figure 3a), the power spectral density for the intervals I, II, III representing vasomotion activity (Figure 3b) and the power spectral density for the intervals IV and V (Figure 3c) were not different between the groups. The relative contributions of intervals I, II, III and IV were not different between the three groups (Figure 3d). The relative contribution of the interval V characterizing the effect of cardiac output on TOI variability was increased in the SS group in comparison with AA and SC groups (SS>SC = AA) (Figure 3d). A representative example of Fast Fourier Transform analysis of the TOI signal variability in three subjects (1 AA, 1 SC and 1 SS patient) is shown on Figure 4.

### Muscle VO2 Correlations and Multivariate Linear Regression Models

In the AA group, muscle VO_2_ was negatively correlated with TOI (r = −0.7; p<0.01) and adipose tissue thickness (r = −0.61; p = 0.01), positively correlated with vasomotion activity (*i.e.,* TOI variability of interval I+II+III; r = 0.69; p<0.01) and tended to be positively correlated with hemoglobin concentration (r = 0.45; p = 0.08). No correlation was observed between muscle VO_2_ and SpO_2_, mFBF or hemorheological parameters in the AA group. To identify factors independently associated with muscle VO_2_, a multivariate linear regression analysis was performed. The multivariate linear regression model included muscle VO_2_ as dependent variable and hemoglobin concentration, TOI, adipose tissue thickness and vasomotion activity as covariates. The overall model was statistically significant (R^2^ = 0.64; p = 0.015) and only TOI remained significantly associated with the muscle VO_2_ (beta = −0.62; p = 0.045).

In the SC group, muscle VO_2_ was positively correlated with mFBF (r = 0.77; p<0.001) and vasomotion activity (r = 0.71; p<0.001), negatively correlated with the RBC disaggregation threshold (r = −0.52; p<0.05) and adipose tissue thickness (r = −0.741; p<0.001), and tended to be negatively correlated with TOI (r = −0.43; p = 0.06). Because the p value was less than 0.20 between RBC aggregation index and muscle VO_2_ (p = 0.16), RBC aggregation index was included in the multivariate linear regression analysis with TOI, mFBF, RBC disaggregation threshold, adipose tissue thickness and vasomotion activity as covariates, and muscle VO_2_ as dependent variable. The overall model was highly statistically significant demonstrating that these variables together account for almost 90% of the variation in muscle VO_2_ (R^2^ = 0.89; p<0.001). Among these variables, adipose tissue thickness (beta = −0.56; p = 0.01), mFBF (beta = 0.39; p = 0.03) and the RBC disaggregation threshold (beta = −0.55; p = 0.03) remained significantly and independently associated with the muscle VO_2_.

For the SS group, we observed a positive correlation between VO_2_ and mFBF (r = 0.67; p<0.01). No correlation was observed between muscle VO_2_ and the other parameters but both RBC elongation index (p = 0.11) and RBC aggregation index (p = 0.13) were included with mFBF as covariates in the multivariate linear regression model to test the independent association with muscle VO_2_. The overall model was statistically significant (R^2^ = 0.47; p = 0.048) and only mFBF remained significantly associated with the muscle VO_2_ (beta = 0.73; p = 0.03).

### Muscle Force and Fatigability

Muscle force and fatigability are summarized in the Table 2. The pre-MVC values were lower in SC and SS groups compared to the AA group. The post-MVC values were lower in the SS group compared to the AA group. Although the post-MVC values were not statistically different between the SC and AA groups, they tended to be lower in the SC patients (p = 0.07). In each of the three groups pre-MVC values were significantly higher than post-MCV values. No difference was observed between the three groups for the RMS pre-MVC and post-MCV. Nevertheless, the two sickle cell groups tended to have a higher RMS pre-MVC than the AA group (p = 0.1). RMS pre-MVC and RMS post-MVC were not different. No difference was observed between the three groups for the percent of force decrease between pre-MVC and post-MCV.

## Discussion

This is the first study investigating muscle microvascular metabolism in the sickle cell population. We demonstrated 1) normal muscle microvascular oxygenation in SC patients but reduced muscle microvascular oxygenation in SS patients when compared with AA control subjects; 2) no difference between AA, SC and SS subjects regarding muscle oxygen consumption or vasomotion activity despite the presence of hemorheological abnormalities in the sickle cell population; 3) muscle microvascular blood flow was significantly higher in the SS group compared to the AA group, and tended to be higher compared with SC patients; 4) a significant association between muscle oxygen consumption and muscle microvascular blood flow in SC and SS patients (and with the RBC disaggregation threshold in SC patients only), whereas muscle oxygen consumption is dependent on muscle microvascular oxygenation in AA control subjects. Local exercise challenge showed 1) a reduced forearm maximal voluntary contraction in SS and SC patients compared to the AA group; 2) similar force decrement after an intermittent handgrip exercise in the three groups.

### Hemorheological Parameters

The observed blood rheological abnormalities of SC and SS groups were in agreement with previous studies [30], [31], with RBC deformability and aggregation being reduced and the RBC disaggregation threshold (*i.e.,* the strength to separate RBC aggregates) being abnormally elevated in the SCD patients in comparison with the control subjects. In non-SCD context, it has been demonstrated that hemorheological abnormalities may alter blood flow in macro- and microcirculation, as well as tissue oxygenation [10], [32], [33]. However, the present study showed that the reduction of muscle microvascular oxygenation in the SS group was not associated with the degree of hemorheological alterations. Moreover, despite the presence of blood hyperviscosity in SC patients, this group had normal muscle microvascular oxygenation. These findings suggest that the muscle microvascular system at rest may adapt to compensate for the RBC rheological disorders and to provide enough oxygen to muscle tissue.

### Methodological Considerations and Limitations

We found no correlation between adipose tissue thickness and muscle microvascular oxygenation and microvascular blood flow. In contrast, a significant correlation was found between adipose tissue thickness and muscle oxygen consumption in AA and SC groups but not in the SS group. Because adipose tissue thickness is a substantial confounder in the measurement of near-infrared parameters, muscle oxygen consumption could have been underestimated in the AA and SC groups. Nevertheless, in this experiment, attention was devoted to make the skinfold thickness very comparable between the three groups. The mean difference between the groups for adipose tissue thickness was 0.1–0.4 mm and was not significant. It was previously shown that the near-infrared light absorbance was decreased of 30% when the thickness of the fat layer increased from 2.5 to 5 mm [19]. We believe that the small difference of adipose tissue thickness between our groups had a limited influence on the mean muscle oxygen consumption of AA and SC groups. Nevertheless, because the value of the adipose tissue thickness ranged from ∼1.4 to 6.7 mm in each group, this parameter was considered in the multivariate linear regressions models to identify factors associated with the local muscle VO_2_ within AA and SC groups. We reported that adipose tissue thickness remained independently associated with muscle oxygen consumption in the SC group, only.

Unfortunately, we were not able to normalize the muscle oxygen consumption by the muscle mass. It may be a limit of the study since a greater number of muscle cells will increase the value of resting muscle oxygen consumption. Nevertheless, it has been clearly demonstrated that SS patient have usually lower lean and fat mass than healthy subjects [34], [35] because of an increased rate of protein turnover [35]. Badallo et al [36] suggested that this higher protein turnover rate could also occur at the muscle level in SS patients. A lower muscle mass in sickle cell patients could result in a greater muscle VO_2_ in SS patients compared to the AA and SC groups. Nevertheless, it is also possible that NIRS signal could be poorly affected by muscle mass since only a limited proportion of the whole muscle size is analyzed: the spatial resolution of the NIRS signal is limited at approximately the half of the interprobes distance (*i.e.,* ∼2 cm) [19]. The impact of muscle mass on muscle oxygen consumption measurement using NIRS device is probably not linear and specific designed protocols to address these issues are needed.

### Muscle Microvascular Metabolism

Despite reduced muscle microvascular oxygenation (in SS group only) and hemorheological alterations, the muscle oxygen consumption in SC and SS patients was not different from the AA group. One may hypothesize that muscle oxygen extraction could be increased in SS patients. The reduced affinity of hemoglobin S for oxygen at low oxygen partial pressure [37] could facilitate the delivery of oxygen to the tissues, thus decreasing muscle microvascular oxygenation but maintaining muscle oxygen consumption to normal level. But, it could also be argued that the reduced affinity of hemoglobin S for oxygen could be offset by an impaired oxygen loading in the lung capillaries. However, although a significant amount of sickle cells (the more dense) are not able to load oxygen as well as than discoid sickle cells [37], the hemoglobin oxygen saturation of the SS group was only modestly decreased. In addition, no correlation between muscle oxygenation or muscle oxygen consumption and arterial hemoglobin oxygen saturation was observed in our study.

The reduction of the muscle microvascular oxygenation in SS patients could be at the origin of the increased muscle blood flow in this group since a decrease of the arterial oxygen content usually results in a rise of blood flow [38]. Ellsworth et al. suggested that RBCs are able to detect a reduction of the hemoglobin oxygen saturation and, in turn, release ATP into the circulation to cause vasodilation and increase blood flow [39]. In addition, one could suggest that the reduced microvascular oxygenation could lower the muscle oxygen consumption leading to an increased glycolytic end-products production, such as adenosine, causing the relaxation of vascular smooth muscle cells and vasodilation [40]. Indeed, the increased muscle microvascular blood flow in SS patients could serve to maintain muscle oxygen consumption to normal level. However, another mechanism can be proposed. The reduced microvascular oxygenation coupled with a normal resting muscle oxygen consumption could indicate that there is slight hypoxia within the muscle which is not sufficient to limit mitochondrial respiration (because of high affinity of cytochrome c oxidase for oxygen) [41]; and 2) the increased microvascular blood flow is a consequence of the slight hypoxia, which would serve to partially reverse the hypoxia, without affecting muscle oxygen consumption, because even moderate hypoxia does not limit resting respiration rate [41].

In the SC group, an independent association was also observed between muscle oxygen consumption and the RBC disaggregation threshold. The elevated RBC disaggregation threshold in SC patients may increase flow resistance at the entry of capillaries where RBCs aggregates need to be fully dispersed before they can be able of entering into, and negotiate, small capillaries [32]. Indeed, high RBC disaggregation threshold could impair muscle perfusion and muscle oxygen consumption. The reasons why this association was observed in the SC group and not in the SS group, despite a comparable RBC disaggregation threshold in the two groups, are unknown. But, one may hypothesize that the higher microvascular blood flow observed in SS patients could break-down the existing RBC aggregates more easily than in SC patients, hence limiting the impact of the elevated RBC disaggregation threshold on the microcirculation of SS patients at the muscle level.

Although vasomotion was positively correlated with muscle oxygen consumption in SC patients (but not in SS patient), the multivariate linear regression failed to demonstrate an independent association between these two parameters. Vasomotion activity is known to be less pronounced in muscle than in other organs such as brain or kidneys where vasomotion is a key regulator of tissue perfusion [40]. Our results suggest that muscle microvascular blood flow is more effective than muscle vasomotion activity to maintain normal resting muscle oxygen consumption in sickle cell patients.

We did not address the role of the autonomic nervous system activity, and more particularly of the sympathetic activity, on the muscle blood flow regulation in this study. It is known that sickle cell patients (mainly SS patients) are characterized by an autonomic imbalance with a loss of heart rate variability [3], [42]. Whether this autonomic imbalance impacts on the regulation of the baseline muscle blood flow in this population is unknown. Nevertheless, the results obtained with the spectral analysis of the NIRS signal demonstrates that interval II (figures 3b and 3d), which depends on the sympathetic activity of the vessel wall, did not differ between SS patients and the two other groups. Indeed, it is highly possible that local vasodilatory substances released by muscles or RBCs have played a role in the positive regulation of muscle blood flow.

### Muscle Force and Fatigability

The short and local handgrip exercise demonstrated a reduction of the maximal voluntary muscle force in SS and SC patients compared to AA group. This result is in accordance with the literature [43]. Although the RMS values of the pre-maximum voluntary contraction were not significantly different between the three groups, they tended to be lower in sickle cell patients compared to the control group (p = 0.1). RMS is an indicator of the spatial and temporal muscle fibers recruitment [44]. Therefore the reduction of the maximal voluntary muscle force in SS and SC patients may be attributable to a lower muscle mass or neural recruitment that could be due to strength deconditionning and physical inactivity. In contrast, the decrease of force after the intermittent handgrip exercise was not different between the three groups suggesting that local muscle fatigability is similar in patients and control subjects. The normal muscle metabolism and the normal or higher local microvascular blood flow observed in sickle cell patients probably participate in maintaining a normal muscle function during a local submaximal exercise. Nevertheless, the microvascular adaptations observed at rest could be of limited impact in SCD patients during longer or more intense physical efforts since the important cardio-respiratory work is not sufficient to compensate for the adverse effects of anemia on tissue oxygen delivery during intense exercise [9], [30], [45].

In conclusion, we demonstrated that sickle cell patients have normal baseline muscle oxygen consumption despite hemorheological alterations and that only SS patients had reduced muscle microvascular oxygenation and increased microvascular blood flow. The increased muscle microvascular blood flow in SS patients could be either a way to compensate for the reduced muscle microvascular oxygenation, hence maintaining muscle oxygen consumption to normal level or a consequence of a slight hypoxia within the muscle, which is not sufficient to limit the muscle oxygen consumption. Altogether, these results suggest that the limited maximal exercise capacity in sickle cell patients is rather due to chronic anemia, cardiorespiratory over-solicitation and/or physical inactivity than muscle metabolism alterations. However, further studies are needed to characterize muscle metabolism at exercise and in most severe patients.
